# Seasonal spreading and transport of buoyant plumes in the shelf off Kochi, South west coast of India- A modeling approach

**DOI:** 10.1038/s41598-019-56103-9

**Published:** 2019-12-27

**Authors:** G. Seena, K. R. Muraleedharan, C. Revichandran, S. Abdul Azeez, Sebin John

**Affiliations:** 1 CSIR-National Institute of Oceanography, Regional Centre-Kochi, Kerala, India; 2 Research Scholar, Bharathidasan University, Tiruchirapalli, India

**Keywords:** Environmental impact, Physical oceanography

## Abstract

We investigated the seasonal spreading and transport of buoyant plume in the shelf off Kochi using Finite Volume Community Ocean Model (FVCOM). The modelled river plume typically consisted of an offshore bulge and a coastal current. The spreading of the bulge extended up to a distance of 19 km from inlet during the summer monsoon to <10 km in the spring inter-monsoon. The Kelvin number varied between 0.1 and 0.9 which revealed that the plume exhibited both the features of small and large scale plumes, resulting in a highly complicated plume pattern. During the southwest monsoon the plume fringe twisted towards the south, while during the northeast monsoon it twisted towards north according to the reversal of monsoonal winds. The fresh water transport with respect to coastal currents varied in accordance with seasonal river discharge such that the value peaked in the wet season and dropped in the dry season. During the non-realistic (no wind) condition the plume initiated barotropic and baroclinic flow, after which it was acted upon by earth’s rotation so that the plume propagated in the direction of Coriolis force (towards north), as geostrophic currents. The model run ‘with wind’ and ‘without wind’ condition revealed that in the shelf off Kochi the plume is transported in accordance with monsoonal winds/currents by nullifying the effect of earth’s rotation. The categorization of plume influenced area and realization of the direction of plume transport can be used for interpreting the dynamically and potentially active zones in the shelf off Kochi.

## Introduction

The fresh water influx and the wave action make the coastal ocean a very dynamic and enriched ecosystem. The low saline estuarine/fresh water which protrudes out of the estuarine/river mouth into the shelf region floats over the high saline oceanic water as a buoyant plume. The outward extension of buoyant plume depends on a large number of factors such as volume of outflow, inlet width, tidal action, wind, currents, local bathymetry, Coriolis acceleration etc., The plume can be separated into two dynamically distinct regions: a bulge near the river mouth and a downstream coastal current^1^. Yankovsky and Chapman^2^ suggested that the dynamics within the bulge are primarily cyclostrophic in nature, i.e., the momentum balance dominated by the pressure gradient, the Coriolis force and the centrifugal force associated with the azimuthal velocity around the bulge. The discharge into the coastal region can produce a strong buoyancy-driven coastal current that drags the low saline water to long distances. Since the fresh water influx has a strong dependence on seasonal rainfall, it drastically affects spatial extension and vertical gradients of the plume. This also has several effects on coastal zone properties such as reduction of salinity, increase in stratification and distribution of parameters like dissolved matters, pollutants, nutrients etc.^3–8^.

Schettini *et al*.^9^ categorized buoyant plumes into riverine and estuarine plumes, based on the amount of mixing occurring before a plume enters into the sea. In riverine plumes, fresh water is directly injected into the shelf region due to heavy river discharge, while in estuarine plumes the fresh water gets mixed with saline water within the estuary and as a result a modified buoyant plume protrudes out into the shelf. The salinity distribution pattern of the freshwater/estuarine plumes has a strong correlation with seasons. The gradients in salinity/density create plume fronts and their span of occurrence depend on the plume dynamics. The disturbance in the plume frontal region may initiate cross frontal mixing through isopycnal layers resulting in the upward movement of nutrient-rich subsurface layers and leading to enhanced productivity of surface waters. A. Jay *et al*.^10^ identified this plume mixing as a key component for explaining the entrainment of nutrients in the sunlit layers and enhanced productivity on the shelf region. The studies by Nash and Moum^11^ revealed that river plumes are one of the sources behind the creation of large-amplitude internal waves in the coastal ocean. The direction of plume propagation is another major factor in establishing the plume behaviour and its environmental impact. The offshore behaviour of the plume can be predicted by calculating Kelvin number as the ratio of source width to Rossby radius of deformation^12^. Thus the plume can be classified into small scale plume (nonlinear discharge which is negligibly affected by earth’s rotation) and large scale plume (linear discharge which is strongly affected by Coriolis force). The plume continuously transports across the shelf and the dynamics associated with it eventually result in the dilution of plume with the ambient saline water^13–15^.

**Figure 1:**
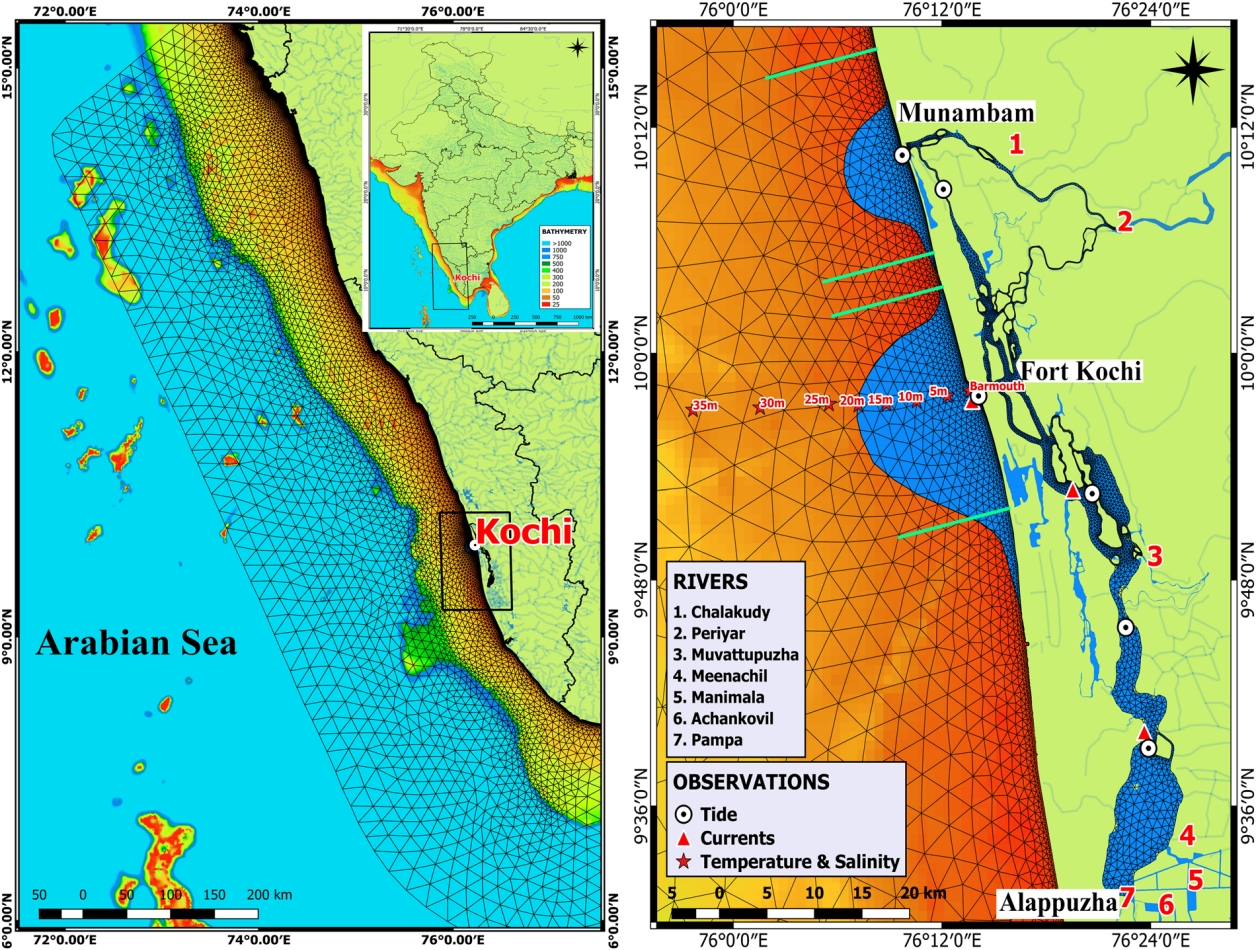
The geographical area covering south west coast of India (left panel) showing finite elemental grid adapted for estuarine-coastal FVCOM model study. Cochin estuary and adjacent coastal waters (right panel) illustrate complex geography where 7 rivers debouch freshwater. Bulging of the plume from the estuary has been conceptually shown over the shelf. *In-situ* observations of the tide, currents and salinity at various location for the validation of the model has shown using symbols. The map was created using QGIS version 2.18.19, QGIS Development Team (2018), QGIS Geographic Information System, Open Source Geospatial Foundation Project available http://qgis.osgeo.org.

**Figure 2:**
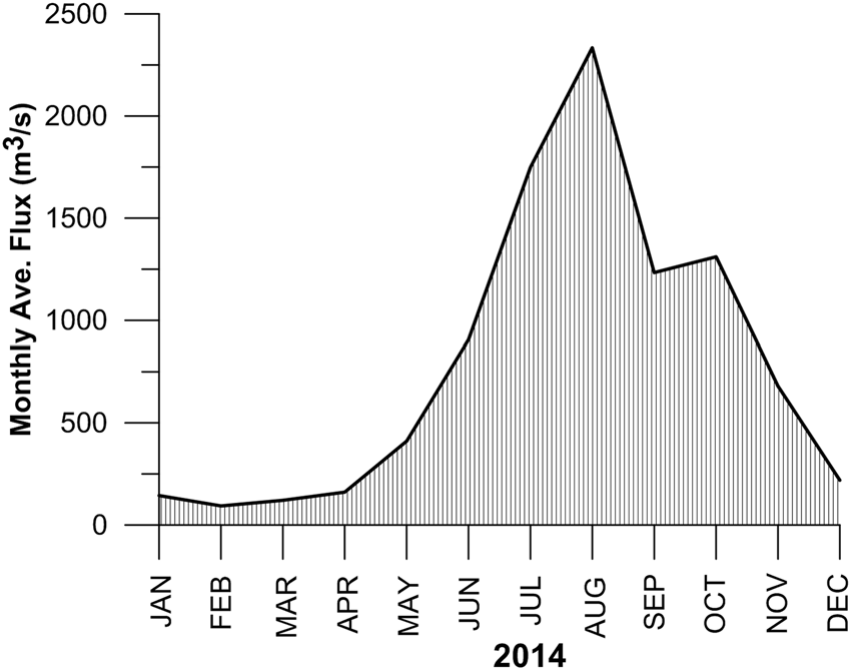
Monthly average river discharge from all the rivers debouching to the Cochin estuary during the year 2014. Heavy discharge can be seen during July-August with a secondary peak discharge during October, while February-April shows the least discharge.

The south west coast of India (SWCI), extending from 8°N to 13°N in the eastern Arabian Sea, is well- known for its intense monsoonal rainfall and seasonally reversing coastal currents (Fig. 1). The largest estuarine system associated with this coastal region is the Cochin estuary (Kochi) that extends from Munambam (10°10′N, 76°15′E) in the north to Alappuzha (09°28′N, 76°25′E) in the south over a length of ~96 km and an area of 256 km^2^. Mixed semidiurnal tide (~1 m) that propagates through the inlets, interact with geomorphology and bathymetry and the freshwater influx from the hinterland rivers cause complex hydrodynamics. The system receives high annual fresh water inflow of 2.2 × 10^10^ m^3^ from rivers Periyar, Chalakudy, Muvattupuzha, Meenachil, Manimala, Pampa and Achankovil^16^. However, 60–70% of the annual river discharge occurs during the summer monsoon months (Fig. 2). Since the estuarine volume of 521 × 10^6^ m^3^ cannot hold the large monsoonal riverine influx, it is further transported as a plume to the shelf- through two major openings: Fort Kochi and Munambam. These out flows generate buoyant structure in the coastal waters and play a key role in the marine ecosystem by influencing stratification, frontogenesis, internal waves, nutrient enrichment and enhanced phytoplankton growth.

The present study is motivated by the need to understand the plume initiated salinity gradients in the shelf off Kochi and to trace the direction of propagation of plume with respect to wind forcing and Coriolis force. As the study region is influenced by the summer monsoon, fall inter-monsoon, winter monsoon and spring inter-monsoon with strong and directionally varying coastal currents, it is imperative to know whether the plume transport is influenced by the coastal current or the earth’s rotation.

## Methodology

Intense rainfalls associated with summer monsoon (June-September) induce freshwater plumes over the shelf off SWCI; while southwesterly winds associated with this season initiate strong coastal currents through the plumes causing quasi-geostrophic dynamics. The seaward expansion of the plume can be characterized by (1) acceleration, resulting from the balance between inertia and buoyancy (gravity) forces; (2) mixing, governed by turbulence due to bottom and interfacial friction and (3) geostrophy, where the balance between Coriolis force and pressure gradients generates an alongshore coastal current, which is scaled by the baroclinic Rossby radius of deformation^17^. The studies require high spatio-temporal resolution data to delineate the processes associated with it and hence calibrated/validated coastal hydrodynamic model was utilized for studying the dynamics of the plume over the shelf off Kochi, SWCI.

### Modelling approach

The Finite Volume Community Ocean Model (FVCOM, v. 4.1) was used to simulate the hydrographic and dynamic nature of the buoyant plumes along the SWCI for a period of one year (2014). FVCOM is an unstructured grid, finite-volume, free surface, three-dimensional primitive equation coastal ocean model that solves the momentum, continuity, temperature, salinity and density equations^18^. The model decomposes primitive equations over unstructured triangular grids, having spatial resolution varying from 20 m (within 10 km area of inlet) to 50 km (in the open ocean) and consist of about 60,000 elements with 21 vertical sigma levels. Global self-consistent, hierarchical high-resolution geography database available at http://www.soest.hawaii.edu/wessel/gshhg/ was utilised for delineating land-water interface, while estuaries and backwaters were incorporated by digitizing LISS-III data. The river discharge for the year 2014 was taken from Central Water Commission (CWC) data set. Surface meteorological forcings such as atmospheric pressure, wind, solar radiation and cloud were taken from European Centre for Medium Weather Forecast (ECMWF). Tides were forced from the open boundary using elevation data created from FES 2014. Temperature, salinity and currents along the open boundary were forced from global Hycom model (http://tds.hycom.org/thredds/catalogs/GLBv0.08/). The most suitable time step for the model was 1 s and the outputs were recorded at every hour.

### Calibration and validation of the estuarine-coastal ocean model

#### *In-situ* measurements

Validation/calibration of the water level and current simulations for the summer monsoon period were carried out using *in-situ* measurements spanning from 24^th^ July-21^st^ August 2010 in the Cochin estuary, while those for fall inter-monsoon were done from 20^th^ September-20^th^ October 2009. Water levels were recorded at 10 minutes interval at 6 different locations (Fig. 1) using SBE-26 plus water level recorder (accuracy of 0.1% of full-scale). Aanderaa RCM-9 current meters were deployed at 4 locations to record speed and direction at 10 minutes interval with an accuracy of ±0.15 cms^−1^ and ±20° respectively. Salinity profiles were obtained from 8 offshore locations (Fig. 1) using SBE-19 plus CTD (conductivity ± 0.001 S/m) during the year 2014.

#### Model validation

The data from *in-situ* environmental observations were used to evaluate model performance. Taylor diagram provides a statistical summary of the model capability to the observed patterns in terms of correlation, centred Root Mean Square Deviation (RMSD) and variance. The correlation between model and observation is given by the azimuthal position, while the distance from the reference point (observations) is a measure of the centered RMSD. Therefore, an ideal model (being in full agreement with observations) is marked by the reference point (0.1) with coordinates ϕ = 0 and radius = 1, which means the correlation coefficient is equal to 1 and the modelled and measured variations have the same amplitude^19,20^.

**Figure 3:**
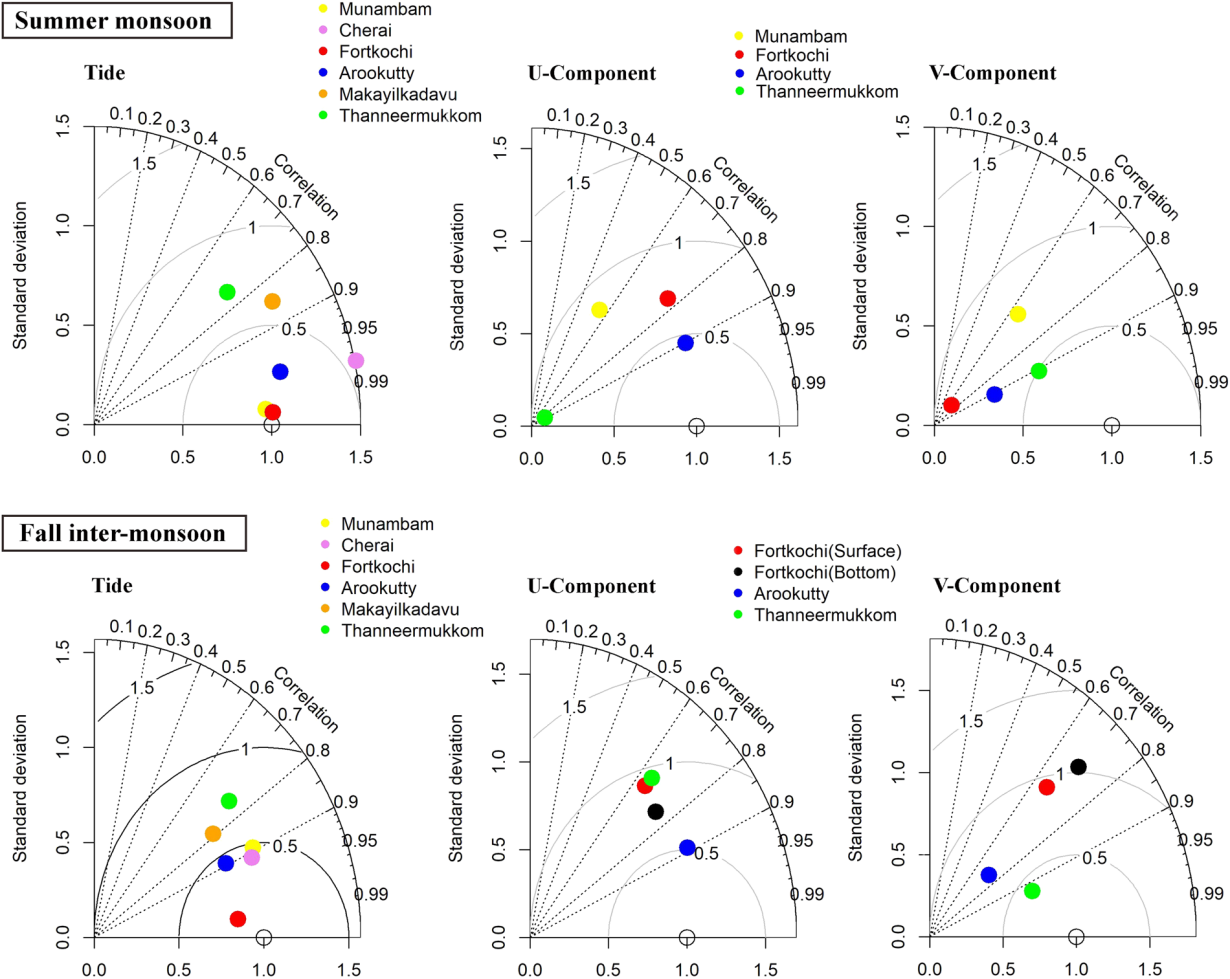
Top panel (Bottom panel) shows the Taylor diagram generated for summer monsoon (Post monsoon). Figures represent statistical significance of the model by comparing simulated tide, U and V components with the observed data from the various locations in the Cochin estuary. Both the seasons, Taylor diagram showed very good correlation (>0.7) of tide and current simulations with a standard deviation <0.7 and low RMSE (<1.0).

During the summer monsoon, the model predicted tidal propagation (Fig. 3) in the estuary was well-matched with the observed tide having a good correlation of >0.85 (except the southernmost station; where the correlation was 0.78), minimum standard deviation (<1) and RMSD (<1). Current measurements strongly influenced by the geometry of the location, depth at which the instrument was deployed and river influx. Although phase of simulated currents was in tandem with observed currents and slight variation in amplitude was noticed. Average correlation of the currents during this period was >0.7 with a standard deviation of <0.7 and RMSD of <1. During the fall inter-monsoon, the river discharge decreased and the tide propagated far inside the estuary, which was well correlated with observation (correlation coefficient <0.8 coefficient). Currents also showed reasonable correlation (>0.7) with low standard deviation (<0.7) and RMSD (<1).

**Figure 4:**
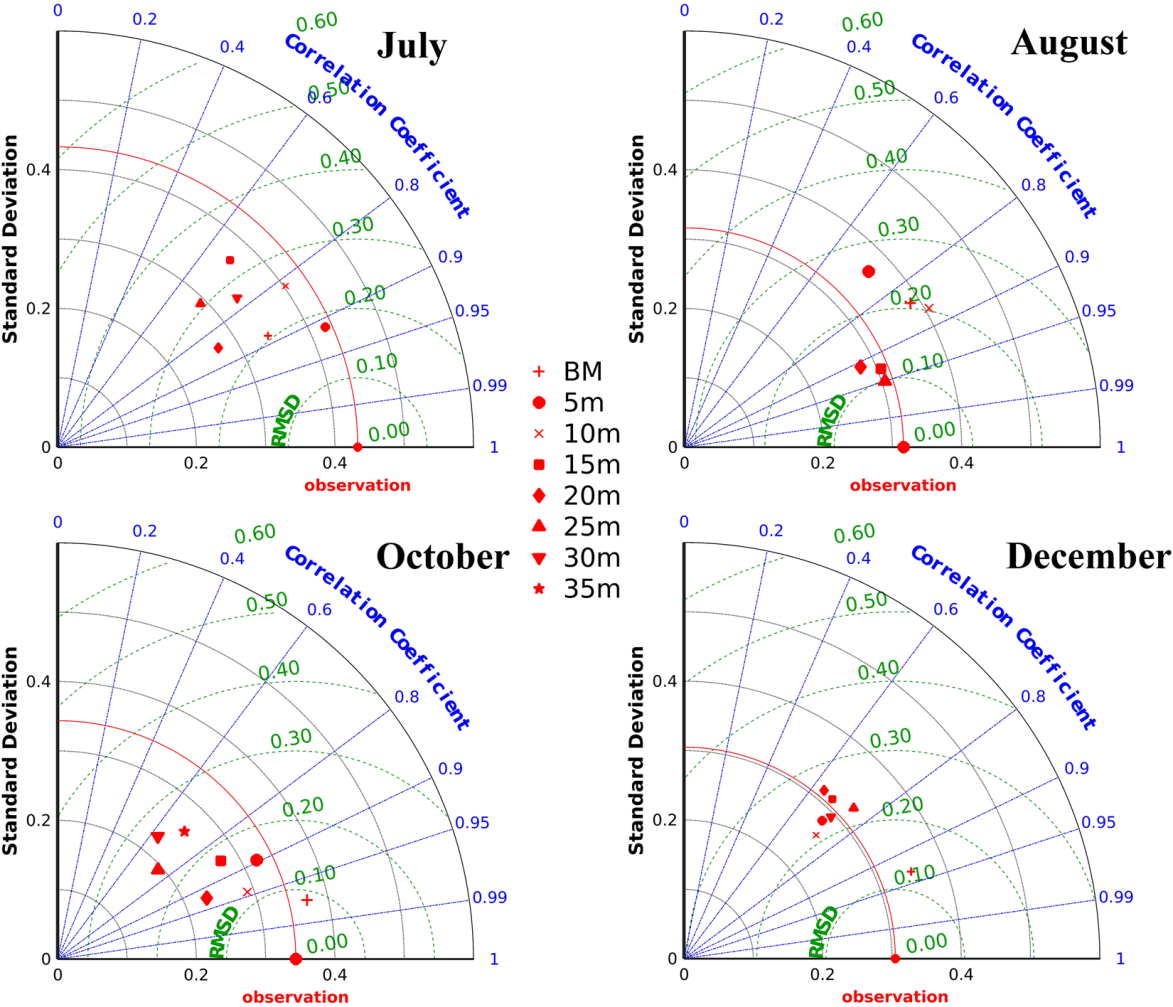
Taylor diagram generated for various months in the year 2014. Figures represent statistical significance of the model by comparing salinity with the observed data from the various location of the shelf off Kochi. Taylor diagram statistically depicts the capability of the model to simulate salinity profiles comparable with the observed profiles over the shelf at various locations.

Salinity variation in the shelf off Kochi was simulated for the year 2014 and was well-matched with *in-situ* measurements. The representative months having large, medium and low riverine flux were subjected to statistical significance (Fig. 4). Throughout the validation, high correction coefficient (≥0.7) was noticed at all stations with a minimum standard deviation and RMSD (<0.3).

#### Quantification of the alongshore transport of buoyant plume and Kelvin number

**Figure 5:**
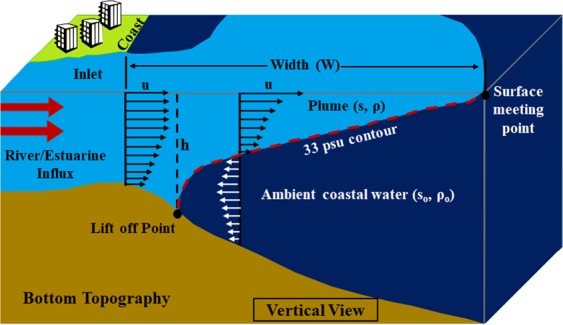
Offshore spreading of the plume over the ambient coastal water detailed the plume width, plume vertical extension, surface meeting point, lit off point etc.,

Garvine^12^ classified buoyant coastal outflow into two categories based on Kelvin number

$$K=W/{R}_{d}$$

where W is the plume width and R_d_ is the baroclinic Rossby radius of deformation

$${R}_{d}=\sqrt{g^{\prime} h}/f$$

where $g^{\prime} =g\frac{\Delta \rho }{{\rho }_{0}}\,$is the reduced gravity, $\Delta \rho ={\rho }_{0}-\rho$, *ρ*_0_ = 1023 kg/m^3^, h is the depth at which 33 psu lift off from the bottom (Fig. 5) and *f* is the Coriolis frequency.

If *K* > 1, the flow is large scale where the plume length far exceeds the width and the flow dynamics is linear. If *K* < 1 the discharge is small scale, the flow dynamics is strongly nonlinear with sharp frontal boundaries and internal hydraulic jumps and the effect of earth rotations are negligibly small^12,21^. If *K* = 1, the system exhibit features of both limiting cases and so tend to be the most dynamically complicated.

**Figure 6:**
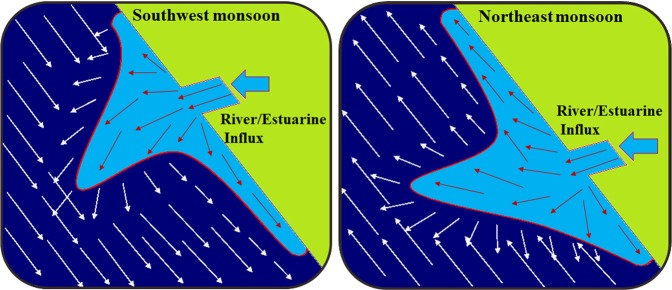
Protrusion of freshwater plume and plume induced currents over the shelf, where quasi-geostrophic currents (red arrow) generated from the plume was modulated by the seasonal coastal currents (white arrow).

While entering the on-shore region the plume developed a growing bulge during summer monsoon while a weakening one during fall inter-monsoon and spring inter-monsoon, where a part of the bulge propagated along with the coastal currents (Fig. 6). Due to the effect of coastal currents, the buoyant plume travelled longer distances with least dilution so that the sign of land efflux was well defined along the coast. The fresh water transport in the coastal current *Q*_*fcc*_ is defined as

$${Q}_{fcc}={\iint }^{}v\frac{\Delta s}{{s}_{0}}d{\rm{A}}$$

where ***v*** is the alongshore velocity, Δ*s* is the salinity difference between the plume and ambient water, *s*_0_ is the ambient salinity (35.5 psu) and the area integral computed over the depth/ cross-shore section of the coastal current.

#### Categorization of plume influenced area

Alves *et al*.^22^ classified the saline water in accordance with the salt content in to oligohaline (0.5–5 psu), mesohaline (5–18 psu), polyhaline (18–30 psu) and euhaline (>30 psu). Here the area of impact of plumes was categorized by calculating the fresh water content, $FF=\frac{{S}_{0}-S}{{S}_{0}}$ where *s*_0_is the reference value of salinity (35.5 psu) and *s*is the salinity of the buoyant plume as 0.5–5 psu, 5–18 psu, 18–30 psu and 30–34 psu. In this study the area of influence of the riverine inflow in the shelf region was identified and categorized using fresh water fraction (FF) and salt content as, fresh water/freshwater plume (FF lies between 0.98–0.86), freshwater plume influenced (0.86–0.49) estuarine plume (0.49–0.15) and estuarine plume influenced (0.15–0.01).

## Result

Oceanography of SWCI dominates by summer monsoon (June-September), winter monsoon (December-February), spring inter-monsoon (March-May) and fall inter-monsoon (October- November). The region is subjected to semi-annual wind reversals associated with the monsoonal cycle that results in elevated biological activity during southwest monsoon (SWM) and northeast monsoon (NEM). However, during fall inter-monsoon and spring inter-monsoon, the high productivity brought down as a result of quiescent wind and poor dynamics. West coast of India is peculiar by its shelf geometry having narrow width at the south and broader at the north where the shelf width off Kochi is about 70 km. The shelf receives a considerable amount of fresh water as plume from westward flowing rivers originating from Ghats and mix with coastal waters under the influence of wind and shelf dynamics. The rate of mixing and direction of transport of these buoyant plumes can be identified and tracked by salinity gradients.

### Classification of the buoyant plume with respect to Kelvin number

Buoyant plume in the shelf region is one of the striking signatures of riverine discharge and the classification with respect to Kelvin number of these mesoscale features throw light on the propagation of buoyant plume in the shelf region. The development of the plume trajectory is highly related to the density gradient, the kinetics acquired within the estuary, the plume width and the Rossby radius of deformation.

The plume features of the shelf off Kochi which determines the value of Kelvin number are drastically varying by the altering seasonal winds and river efflux (Figs. 2 & 6). Table 1 highlight that the plume width varied from 3.5 km-14.4 km in Fort Kochi inlet and from 0 km-15.6 km in Munambam inlet. Kelvin number of Fort Kochi inlet during the summer monsoon was almost constant (0.3), while that of Munambam inlet varied from 0.2–0.5. Kelvin number reached the highest range (0.7–0.9) during the winter monsoon and dropped to a lower value (0.1) during the spring inter-monsoon in both the inlets. Since the Kelvin number was in the range of 0.1–1 (~1) during all the seasons, the existence of a highly complicated plume pattern in the shelf off Kochi could be established.

**Table 1:** Monthly variation of plume width, Rossby radius of deformation and Kelvin number at Fort Kochi and Munambam inlet during ‘wind on’ condition.

Month	Fort Kochi			Munambam		
	W(m)	R_d_(m)	K	W(m)	R_d_(m)	K
January	6444.6	7069.45	0.91	247	2578	0.096
February	3541.1	6245.1	0.57	0	NA	NA
March	3538	6676.3	0.53	2368	4639.1	0.51
April	4763	17799.1	0.27	2368	11889.8	0.20
May	6447.6	27566.1	0.23	1500	12190.2	0.12
June	9912.4	32218.3	0.31	4641	18450.3	0.25
July	14,430	42272.5	0.34	10701	42202.5	0.25
August	12,818	33827.3	0.38	10701	21970.3	0.49
September	9912.4	30387.8	0.33	2955.3	19179.8	0.15
October	14,439	35164.5	0.41	15572	24727.3	0.63
November	14,439	25870.7	0.56	8836.6	13233.2	0.66
December	9912.4	13369.7	0.74	5923	8184.6	0.72

### Spatial distribution of salinity gradients in the shelf and realisation of aspects behind plume propagation

The paper details the extension of plume towards continental shelf off Kochi during the year 2014. Salinity is the prominent physical parameter of oceanic water that endures gradual gradients due to the influence of riverine efflux. Since the region is strongly influenced by the seasonal features and the river efflux, hence the salinity distribution also varied with respect to the season.

**Figure 7:**
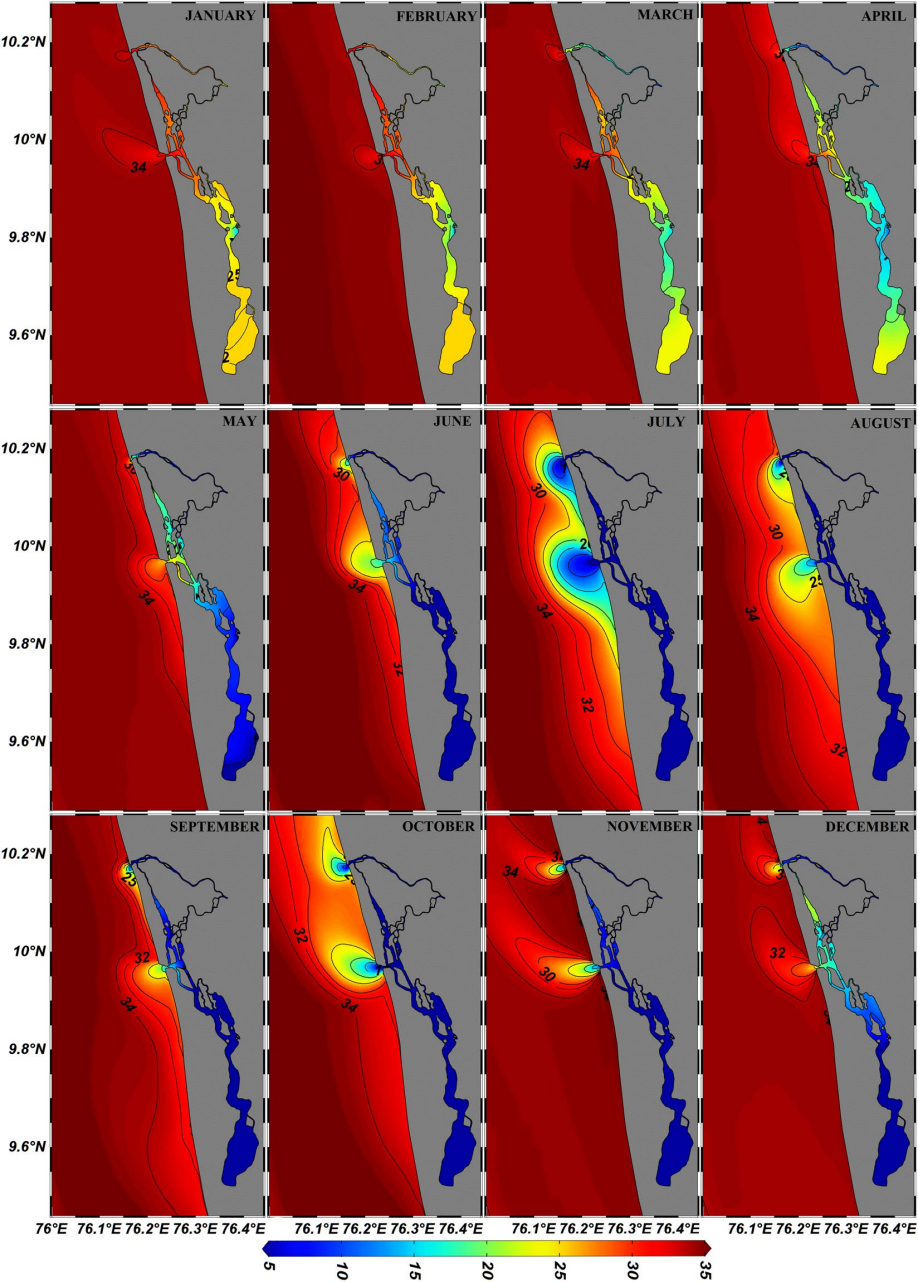
Monthly distribution of freshwater/estuarine plume during ‘wind on’ condition. Large freshwater discharge during summer monsoon showed substantial bulging of the plume, which further modulated by the coastal currents, while least discharge during spring inter-monsoon had the least influence on the coastal waters. The plume bulge twisted towards north and south in accordance with coastal currents.

Summer monsoon is dynamically the most active season which has a great influence on the Arabian sea. The intense precipitation during the SWM and the cumulative discharge of seven rivers (1749 m^3^/s in July) diluted the salinity to a great extent. The freshwater efflux decreased the salinity (Fig. 7) in both the inlets as low as 5 psu and this further protruded out to the shelf forming a ‘pool’ of low saline water over the ambient water (35.5 psu). The salinity of about 7 psu was noticed at a distance of 5 km offshore from the Fort Kochi inlet and 1 km from Munambam inlet. Spreading and intermittent mixing of fresh water over the shelf lowered the salinity of the shelf waters to 18 psu (11 km from Fort Kochi inlet and 5 km from Munambam inlet). The influence of plume was identified (30–34 psu) up to a distance of 19 km from Fort Kochi inlet and 13 km from Munambam inlet. During the season the summer monsoon current flows southward, that affects the transport of buoyant plume. As the Indian coast lies in the northern hemisphere, the buoyant plume is supposed to twist towards the right of its propagation. But during monsoon due to the influence of SWM currents the plume fringe twisted towards the left of its path (Fig. 7). These strong coastal currents not only altered the direction of propagation but also altered the offshore extension of the plume. The month-wise fresh water transport in the coastal current throughout the year (2014) at a distance of 10 km (a region away from plume bulge) towards south and north from both the inlets (Fig. 1, Table 2), quantified the plume propagation with respect to the season. The highest transport (Q_fcc_ = 1731.9 m^3^/s) was noticed in the month of July due to the enhanced fresh water discharge from the estuary and also due to the strong coastal current.

**Table 2:** Quasi-geostrophic currents induced by the plume and associated transport through 10 km north and south of the bulge axis during ‘wind on’ condition.

Month	Q_fcc_ (m^3/^s)			
	Fort Kochi (10 km)		Munambam (10 km)	
	South	North	South	North
January	0	0	0	0
February	0	0	0	0
March	0	0	0	0
April	0	6.16	15.37	13.64
May	202.06	37.68	58.07	0
June	97.1	398.5	380.5	143.73
July	1308	1731.9	1721.3	1269.4
August	1165.9	672.9	622.2	146.1
September	530.7	139.8	150.7	0
October	63.29	426.9	458.1	901
November	0	182.97	62	0
December	0	0	0	0

The summer monsoon is trailed by fall inter-monsoon where the SWM wind weakens and the precipitation decreases, leading to reduced riverine discharge. Even though the total contribution of the rivers to the estuary decreased to 1312 m^3^/s per day (October), the salinity in the inlets (5 psu at Fort Kochi inlet and 0.63 psu at Munambam inlet) decipher the presence of fresh water within the estuary (Fig. 7). The intrusion of the fresh water into the oceanic water decreased the salinity to 18 psu, at a distance of 3 km from Fort Kochi inlet and 1 km from Munambam inlet. When compared with summer monsoon, the offshore extension of plume (salinity of 34 psu) in the fall inter-monsoon period increased up to a distance of 21 km from both the inlets. This is mainly due to the diminishing coastal currents, where the eastern Arabian Sea is in a transitional state from SWM to NEM. The offshore protrusion of the plume slightly twisted towards north under quasi-geostrophic balance, where the fresh water transport in the coastal current ceased to 63.29 m^3^/s (October) at 10 km south of Fort Kochi inlet (Table 2). The northeast wind strengthened during the winter monsoon period along the coast off Kochi and the estuary linked with it was refreshed by an average river discharge of 219 m^3^/s (December). These riverine effluxes were acted upon by tides within the estuary, mixed with the oceanic water and created an estuarine plume of decreased salinity (20–30 psu). Even though the fresh water efflux was lower during this period, the dynamics of the plume decreased the salinity of shelf waters to 32 psu (Fig. 7) up to a distance of 3–4 km and to 34 psu up to a distance of 16–19 km from Fort Kochi and Munamban inlets respectively. The plume fringe twisted towards north in accordance with the reversing coastal currents and the salinity contours were very less in number. Even though the strength of coastal current was advancing, the fresh water transport through the coastal currents at a distance of 10 km south/north from the inlets was negligibly small due to lesser riverine efflux.

The spring inter-monsoon which followed the winter monsoon where the wind blows very quiescently and the river discharge was very less compared with other season (121 m^3^/s in the month of March). The main feature of the season was the absence of fresh water over the shelf while spreading and mixing of the buoyant plume can be identified by the decreased salinity of 34 psu at a distance of 5–10 km from the inlets (Fig. 7). The magnitude of current also ceased so that the offshore extension of buoyant plume tapered.

### Experimental analysis of Kelvin number and plume propagation during ‘no wind’ condition

As the shelf off Kochi lies in the northern hemisphere, the plume fringe was expected to twist towards north due to earth’s rotation. However, in our studies, it is clear that the plume fringe turned towards south/north (summer monsoon/winter monsoon) direction in accordance with the seasonally reversing winds. In order to isolate the effects behind plume twisting, we conducted an experiment with the help of FVCOM by nullifying the influence of wind-driven currents.

**Figure 8:**
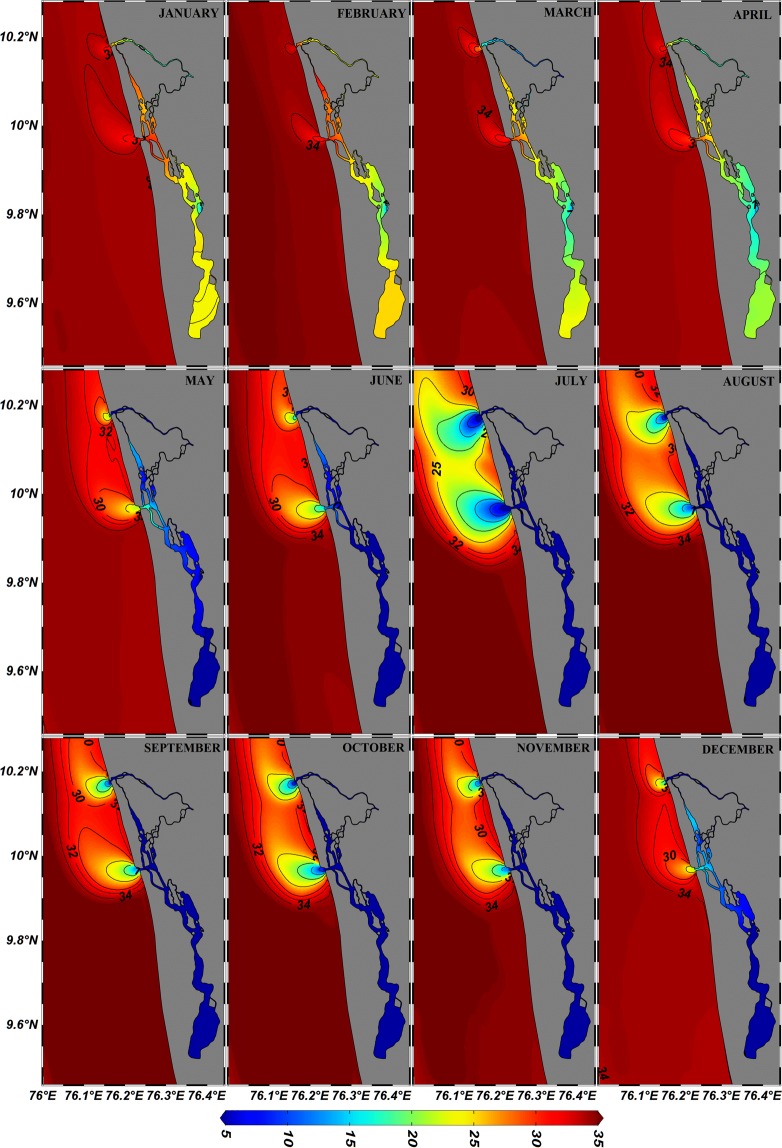
Monthly distribution of freshwater/estuarine plume during ‘wind off’ condition. Freshwater/estuarine plumes generated over the shelf water always showed a northward movement under geostrophic balance.

The experimental study revealed that the plume propagated northward throughout the year independent of the seasons (Fig. 8). During the summer monsoon, the offshore spreading (bulging) of the plume was larger than that in the realistic (natural) situation and this could be correlated to the absence of wind-driven dynamics. Regardless of the seasons, it was observed that the variation in Kelvin number was (0.4–0.7) marginal (Table 3). The experimental situation favoured the effect of earth’s rotation on plume propagation such that after leaving the confines of the estuary the plume always twisted to the right of its propagation. The pressure gradient force (barotropic and baroclinic flow) and Coriolis force were the major parameters responsible for the plume transport as geostrophic currents, where the dilution of the plume was considerably less than that of realistic condition. On quantification of the plume transport with respect to geostrophic currents (Table 4), it was noted that the fresh water transport at 10 km south of Fort Kochi inlet was negligible throughout the year (Q_fgc_ ~0), except in July which was due to heavy river efflux. This indicated that the plume always twisted towards north due to the earth’s rotation. The peculiarity of plume transport in the direction of Kelvin wave was reflected in the increased value of Q_fgc_ at 10 km north of both the inlets compared to the respective 10 km south and the values gradually descended to zero in accordance with the decreasing river efflux. This further confirms that throughout the year, the plume twists towards north in accordance with earth’s rotation, unless and until an opposite strong current overcomes this geostrophic flow.

**Table 3:** Monthly variation of plume width, Rossby radius of deformation and Kelvin number at Fort Kochi inlet during ‘wind off’ condition.

Month	Fort Kochi			Munambam		
	W(m)	R_d_(m)	K	W(m)	R_d_(m)	K
January	6447.6	9334.7	0.69	2955.3	4919	0.6
February	4041.2	6758.9	0.6	1043	2457	0.42
March	4763.2	7422.7	0.64	3560.6	5678	0.63
April	6444.3	12772.1	0.51	2368	5032	0.47
May	11407	25800.4	0.44	7173.7	10812	0.66
June	14439	28727.2	0.5	10701	16836	0.64
July	20790	47268	0.44	19005	33314	0.57
August	17049	33091	0.52	15572	24372	0.64
September	14439	28825	0.5	10701	15051	0.71
October	14439	35455	0.41	13152	19737	0.67
November	14439	27132	0.53	10701	15383	0.69
December	9912.4	16852	0.59	5923	11525	0.51

**Table 4:** Quasi-geostrophic currents induced by the plume and associated transport through 10 km north and south of the bulge axis during ‘wind off’ condition.

Month	Q_fgc_ (m^3^/s)			
	Fort Kochi (10 km)		Munambam (10 km)	
	South	North	South	North
January	0	0	0	0
February	0	0	0	0
March	0	0	0	0
April	0	0	0	0
May	0	95	84	123.8
June	0	175	155.4	217.3
July	21.41	1037	1154	1441
August	0	363.2	365.9	581.2
September	0	179.2	170	241.8
October	0	283.2	280.1	491.8
November	0	203.4	190.5	309.4
December	0	50.4	41.1	74

### Categorization of plume influenced area and its seasonal spreading

The categorization of plume influenced area is very important to envisage the seasonal spreading of plume for a better understanding of shelf ecology. The plume plays an inevitable role in the physical, biogeochemical and ecological functioning of the shelf region. Along with the fresh/estuarine water, the buoyant plume contains nutrients, sediments, pollutants and other constituents^23–26^. All these constituents which lie in the photic zone of the dynamically active shelf region will ultimately influence the biological production (lower to upper trophic levels). One of the main interesting features such as the Potential Fishery Zones (PFZ) along the SWCI is found to be recurrent nearer to the inlets where the terrestrial outputs meet the shelf ambient waters as a buoyant plume. Kripa *et al*.^27^ have opined that the nearshore regions of the Arabian Sea off Kerala with depths less than 50 m occurred more in the PFZ advisory maps than in the mid-continental shelf region and the continental slope. They have also concluded that river runoff plays a critical role in developing PFZs. Therefore, it is important to study the plume influenced area and its seasonal spreading for better understanding and the utilization of living resources.

**Figure 9:**
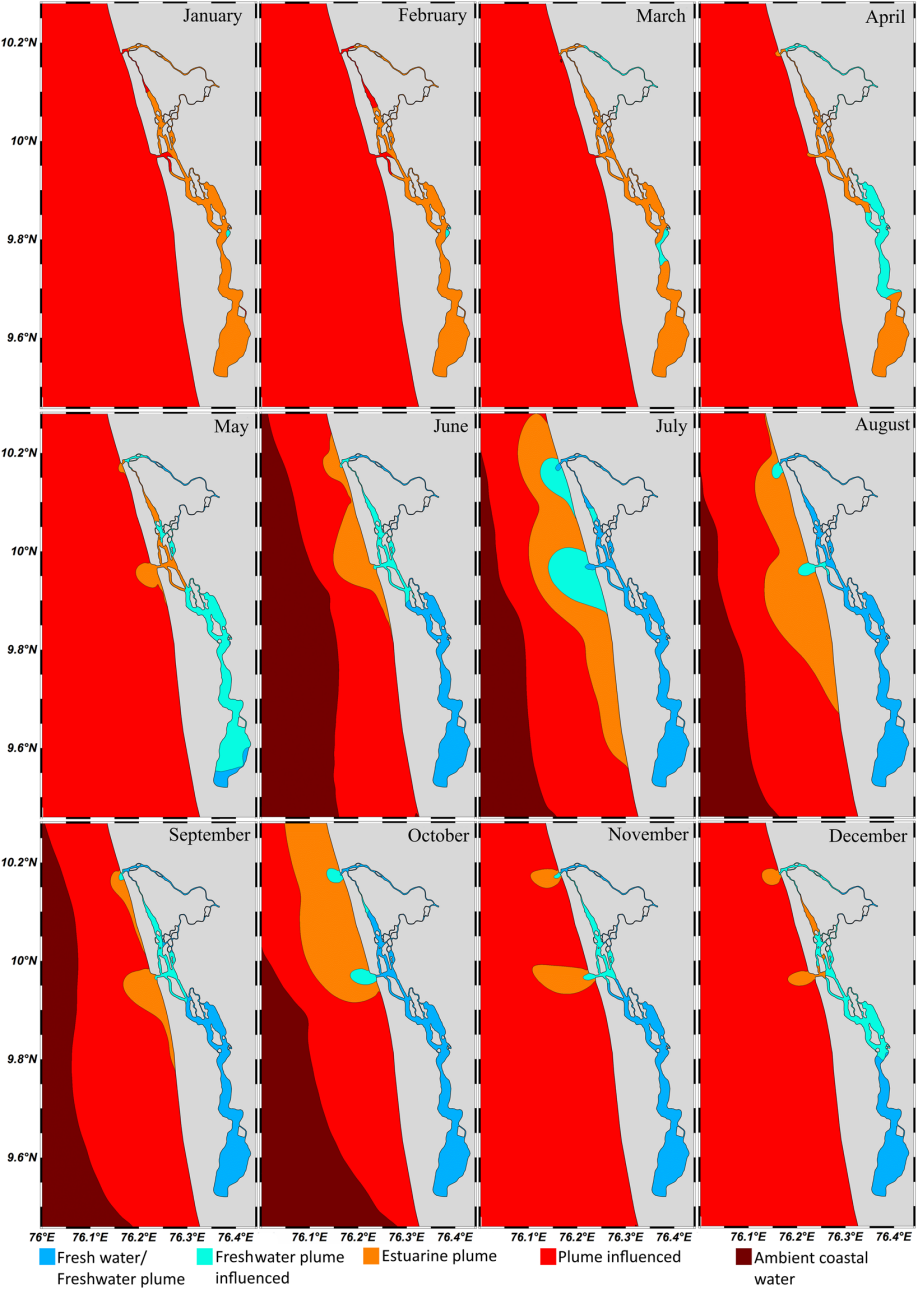
Categorization of plume influenced area and its seasonal spreading in the shelf off Kochi during various months according to salinity and freshwater fraction method.

Summer monsoon is the freshening period of the estuary where the enhanced riverine efflux completely occupies the estuary and the sign of the fresh water efflux was clearly visible in the inlets. The terrestrial flow having FF of about 0.98–0.86 floated over the saline water as freshwater plume (July) and occupied an area of about 2.5 km^2^ nearer to the Fort Kochi inlet and 1.4 km^2^ nearer to the Munambam inlet (Fig. 9). Spreading and mixing of the freshwater plume by the prevailing coastal currents diluted the saline water formed the freshwater plume influenced area (FF between 0.86 and 0.49) and extended about 114.43 km^2^ and 41.88 km^2^ around both the inlets (Fort Kochi and Munambam respectively). The buoyant plume of both inlets merged due to the strong southward flowing coastal currents so that the estuarine plume (FF between 0.49 and 0.15) floated over an area of 495.72 km^2^ in the shelf region with two bulged portion in respect to the two inlets and a propagating nose, that pointed towards the south.

Fall inter-monsoon was featured by the decreased precipitation and diminishing coastal currents. Even though there was declined precipitation, the estuary was completely freshened by the riverine efflux having FF of 0.98–0.86. One striking feature noticed was the total absence of fresh water within the Fort Kochi inlet due to the estuarine dynamics while at the Munambum inlet there was a small protrusion of fresh water as freshwater plume into the shelf (0.01 km^2^). This fresh water projection can be attributed to the major contribution (70%) by the river Periyar (the largest river in Kerala) and shallow bathymetry(∼7 m) of the Munambam inlet. The freshwater plume influenced area in both the inlets (Fig. 9) was less (Fort Kochi inlet-16.31 km^2^ and Munambam inlet-9.07 km^2^) when compared to summer monsoon. Another uniqueness observed in fall inter-monsoon period was the increased offshore extension of estuarine plume (FF between 0.49 and 0.15) to an area of 707.48 km^2^, which was much higher than that in the summer monsoon. This increased spatial extension was mainly due to the cumulative river discharge with quiescent wind so that the plume spread to larger area with least dilution.

Winter monsoon is the season where there is a deficit of fresh water near to the inlets due to intermittent rainfall. This had augmented the nonexistence of fresh water in the vicinity of Fort Kochi inlet and Munambam inlet. A small projection of freshwater plume influenced area was visible (0.11 km^2^) in Munambam inlet due to the efflux from the river Periyar. The outward bulge having an area of 15.81 km^2^ and 12.76 km^2^ near to Fort Kochi and Munambam respectively was due to the presence of estuarine plume over the shelf water (Fig. 9). The absence of the propagating part of the bulge indicates the complete dilution of estuarine plume within this area. Spring inter- monsoon is the season where there is lack of fresh water within the estuary. The one and only indication of fresh water in the form of estuarine plume was near to the Munambam inlet which occupies only an area of about 0.12 km^2^.

## Discussion

The paper thoroughly analyzes the salinity gradients of the shelf off Kochi due to fresh water efflux and plume spreading with respect to different seasons. The region is influenced by dry and wet seasons and accordingly the quantity of river discharge into the estuary varied from 1749 m^3^/s (July) during summer monsoon to 121 m^3^/s (March) during spring inter-monsoon. The annual salinity contours in the shelf region (Fig. 7) were much concentrated during the summer monsoon and widely apart during the winter monsoon. Also, in accordance with the monthly river discharge, the salinity of ambient water varied in each season which leads to the spreading of plume of different dimensions.

The estuary gets flooded many times^16^ during summer monsoon due to downpour and through heavy river discharge (Fig. 2), which brought down the average salinity of transient water to the shelf to <5 psu. This also resulted in an offshore flow of fresh water as freshwater plume (FF between 0.86 and 0.98) to an area of 2.5 km^2^ near Fort Kochi inlet and 1.4 km^2^ near Munambam inlet (Fig. 9). Honer *et al*.^21^ opined that the distinguishing dynamical feature of the buoyant plume is the horizontal advection of fresh/estuarine water from the source points that define the shape and character of the plume, where the dilution of the plume is mainly controlled by vertical mixing. In accordance with plume dilution towards the shelf the salinity of shelf water decreased and the area occupied by the plume varied. The plume intially directed towards off shore and formed the bulged portion and a part of which initiated the buoyancy driven coastal current. The bouyancy driven coastal currents was influenced by earth’s rotation^28,29^ and also by prevailing coastal currents^14^.

In realistic condition, during the summer monsoon even though the region lies in the northern hemisphere, the plume was transported towards the left of its propagation due to the prevailing strong SWM currents (Figs. 6 & 7). In order to identify the factors responsible for the direction of propagation of the plume, the Kelvin number (Table 1) was calculated. Since the Kelvin number ranged between 0.1 and 0.98 (≤1) it could be inferred that the plume exhibited both the features of small and large scale plumes. The lower Kelvin number obtained for summer monsoon months indicated that the effect of SWM current on the plume was stronger than the earth’s rotation. Under this condition the plume fringe twisted towards south against the inherent geostrophic flow, ultimately resulting in the propagation of plume in the southward direction. The perimeter flow on the seaward side of the bulge contained both recently discharged river water and “older” recirculating water^14^. The higher values of Q_fcc_ (summer monsoon) at 10 km south and lower values at 10 km north in both the inlets could be attributed to the strength of SWM currents (Table 2).

The gradual decline in river discharge during fall inter-monsoon compared to summer monsoon was due to the receding SWM and reduced river efflux (Fig. 2). However, the relatively higher river discharge observed during October could be attributed to the early winter monsoon spell. The effect of plume towards offshore was about 21 km from the inlets (plume decreased the ocean salinity to 34 psu), which was higher than that in the summer monsoon (13-19 km) due to weakening of SWM winds and currents. Wind influences the transport and mixing of the plume in two ways: direct vertical mixing and wind straining^30^, where the direct vertical mixing enhances the vertical mixing of water column/decreases the stratification (increases plume dilution) whereas wind straining either decreases or increases stratification in response to wind direction and wind length. In the present case, the wind direction was the same as that of the extension of the plume (in the direction of geostrophic currents) which reinforce the stratification and enhance the extension of the plume. The direction of coastal currents due to winds and the plume induced geostrophic currents were in the same direction (towards north) so that the plume fringe twisted towards north (Figs. 7 & 9) which also reflected in the increased value of Kelvin number compared to summer monsoon. As the plume propagated in northward direction, the quantity of fresh water transport with respect to coastal currents was more in 10 km north of both the inlets than 10 km south (Table 2).

NEM winds during the winter monsoon were flowing in the direction of Kelvin wave and hence the plume always propagated towards the north. During this period the terrestrial output ceased to 219 m^3^/s (December) so that the salinity contours were very less in number (Fig. 7). In December, even though the southern part of the estuary was filled with fresh water, due to mixing and dilution within the estuary which lead to the formation of estuarine plume having FF between 0.15 and 0.49 nearer to the inlets. The outward bulge of the plume (Fig. 9) was confined to 10–15 km^2^ area in the shelf region and the plume fringe twisted towards the north (Figs. 6 & 9). The negligible value of fresh water transport with the coastal current at 10 km north and south of both the inlets (Table 2) highlights that the buoyant plume was completely diluted within this area.

The features of spring inter-monsoon were the lower river discharge (121 m^3^/s in March) and weakened monsoonal winds. The offshore extension of buoyant plume tapered due to lower river discharge and estuarine dynamics (Fig. 7). The prevailing quiescent winds and higher Kelvin number (0.5 in March) favoured the propagation of the plume in the direction of Kelvin wave (Table 3). The quantity of fresh water transport with respect to currents was nil at 10 km north and south of both the inlets and gradually reached higher values (Table 2) in response to the initiation of SWM winds and precipitation.

In order to understand the effect of earth’s rotation on plume propagation, the model was run in the ‘no wind’ condition by nullifying the effect of SWM and NEM currents. The role of earth’s rotation on plume twisting and propagation were well established in Fig. 8, where throughout all the seasons the plume was twisted towards the right (north) of its propagation. Once the plume protruded out from source point/inlet, barotropic and baroclinic flow generated and after which it was acted upon by earth’s rotation so that the plume propagated in the direction of Kelvin wave as geostrophic current. The minimum variability in the Kelvin number (Table 3) during the non-realistic condition (0.4–0.7) with the realistic condition (0.1–0.9) revealed that wind and wind-driven currents during the monsoon played a major role in the structuring of the plume bulge and its transport. During the summer monsoon the plume width and the Rossby radius of deformation increased while during the winter monsoon plume width and the Rossby radius of deformation decreased ultimately resulting in a non-fluctuating Kelvin number. The fresh water transport during ‘wind off’ condition as geostrophic currents (Table 4) clarified that the plume was transported more towards the right of its propagation and also strongly correlated with seasonal discharge.

The quantification of the role of monsoonal forcings and earth’s rotation (Table 5) on plume spreading (size) divulged that during the fall inter-monsoon (October), even though the precipitation was less than the summer monsoon, the spreading of the plume was advanced in the shelf region. This was mainly due to the combined effect of monsoonal winds and earth’s rotation in the same direction. In the non-realistic condition, the plume size was comparatively more than that in the realistic case due to the absence of wind-driven dynamics. The higher value of correlation coefficient during ‘wind on’ and ‘wind off’ condition (≥0.88) revealed that the change in the plume size was perfectly consistent with the river discharge.

**Table 5:** The correlation coefficient between plume spreading (size) and river discharge due to monsoonal forcing and earth’s rotation.

Month	River discharge (m^3^/s)	Plume spreading (km^2^)							
		Wind on				Wind off			
		FK	MB	Total	CC	FK	MB	Total	CC
January	116.81 ± 10.24	8.92	0.1	9.02	0.88 (P-value 0.0005)	22.95	4.72	27.67	0.90 (P-value 0.0002)
February	93.79 ± 6.69	3.64	0	3.64		9.26	0.64	9.9	
March	120.65 ± 6.42	7.22	2.41	9.63		9.65	9.37	19.02	
April	162.1 ± 13.81	80.6 *		80.6		33	4.61	37.61	
May	410.9 ± 32.49	129.12 *		129.12		405.26 *		405.26	
June	906.6 ± 117.73	348.21 *		348.21		569.68 *		569.68	
July	1749.08 ± 147.84	1007.29 *		1007.29		1523.6 *		1523.6	
August	2333.62 ± 245.68	1132.44 *		1132.44		1052.48 *		1052.48	
September	1233.51 ± 177.93	339.06 *		339.06		713.15 *		713.15	
October	1312.03 ± 58.21	1355.98 *		1355.98		807.81 *		807.81	
November	679.81 ± 70.67	135.75	40.66	176.41		674.13 *		674.13	
December	218.68 ± 11.99	68.16	29.99	98.15		336.11 *		336.11	

FK-Fort KochiMB-MunambamCC-Correlation Coefficient.

*Buoyant plumes of Fort Kochi and Munambam were merged.

The presence of plume as observed in this study has a great influence on the shelf salinity and temperature. Also, the terrestrial runoff carries a large amount of organic and inorganic loads and debris in particulate and dissolved forms to the shelf, which increases the nutrient concentration of the water column to sustain good biota. Kripa *et al*.^27^ have opined that river runoff and rainfall are the major factors in developing the PFZs and the fewer numbers of PFZs in the southern part of Kerala is mainly due to limited river discharge. According to J. Dagg *et al*.^31^ mixing of the buoyant plume with oceanic water dilutes riverine coloured dissolved organic matter and also enhances light penetration. This points to the fact that plume influenced areas and their dynamics have a major impact on the physical and optical environment of the shelf region. Our studies on the seasonal variations in salinity gradients clearly mapped the plume influenced areas, direction of propagation of plume and the effects of earth’s rotation, eventually highlighting the dynamically and potentially active zones in the shelf off Kochi.

## Conclusion

The shelf off Kochi (study area) is connected to the second largest estuary (Cochin estuary) in India, which always refreshes the coast with immense fresh water flux from seven hinterland rivers (2.2 × 10^10^ m^3^/year). The fresh water flux after leaving the confines of the estuary floats over the ambient saline shelf waters as a “PLUME”. The spreading and offshore extension of plume concurred with the seasonal river efflux and the estuarine dynamics. The well-validated model studies (FVCOM) mapped the plume pattern (salinity gradients) in the shelf and its transport with respect to seasonally reversing winds. The plume descended through the vents, twisted in response to the monsoonal winds and the earth’s rotation. During SWM the plume twisted towards the south, while during NEM it twisted towards north in accordance with the reversing monsoonal winds. The fresh water transport with respect to coastal currents varied in relation with the seasonal river discharge such that the value peaked during the wet season and dropped during the dry season. The Kelvin number was close to 1 exhibiting both the features of small and large scale plumes. During the non-realistic (no wind) condition, the plume initiated barotropic and baroclinic flow and after which it was acted upon by earth’s rotation so that the plume propagated in the direction of Coriolis force (towards north) in the form of geostrophic currents. The fresh water transport with respect to geostrophic currents was always higher in the northern part of the inlets depicting the transport of plume in the direction of Kelvin wave. The model run ‘with wind’ and ‘without wind’ condition revealed that in the shelf off Kochi, the plume was transported in accordance with monsoonal wind-driven currents by nullifying the effect of earth’s rotation. The striking signature of riverine efflux in the shelf off Kochi is the gradients in salinity which create plume fronts. The vertical mixing of low saline nutrient-rich buoyant water with the ambient oceanic water in the frontal region increases the production to a great extent such that the plume influenced areas ultimately serve as the dynamically and potentially actives zones of the coastal region.
